# Magnetically Aligned Ultrafine Cobalt Embedded 3D Porous Carbon Metamaterial by One‐Step Ultrafast Laser Direct Writing

**DOI:** 10.1002/advs.202102477

**Published:** 2021-11-01

**Authors:** Jin Xu, Ruoxing Wang, Haoqing Jiang, Xingtao Liu, Licong An, Shengyu Jin, Biwei Deng, Wenzhuo Wu, Gary J. Cheng

**Affiliations:** ^1^ School of Industrial Engineering Purdue University West Lafayette IN 47906 USA

**Keywords:** laser direct writing, magnetic field alignment, metal–organic frameworks, metamaterials, water splitting

## Abstract

Spatial manipulation of nanoparticles (NPs) in a controlled manner is critical for the fabrication of 3D hybrid materials with unique functions. However, traditional fabrication methods such as electron‐beam lithography and stereolithography are usually costly and time‐consuming, precluding their production on a large scale. Herein, for the first time the ultrafast laser direct writing is combined with external magnetic field (MF) to massively produce graphene‐coated ultrafine cobalt nanoparticles supported on 3D porous carbon using metal–organic framework crystals as precursors (5 × 5 cm^2^ with 10 s). The MF‐confined picosecond laser scribing not only reduces the metal ions rapidly but also aligns the NPs in ultrafine and evenly distributed order (from 7.82 ± 2.37 to 3.80 ± 0.84 nm). ≈400% increment of N‐Q species within N compositionis also found as the result of the special MF‐induced laser plasma plume. (). The importance of MF is further exmined by electrochemical water‐splitting tests. Significant overpotential improvements of 90 and 150 mV for oxygen evolution reaction and hydrogen evolution reaction are observed, respectively, owing to the MF‐induced alignment of the NPs and controlled elemental compositions. This work provides a general bottom‐up approach for the synthesis of metamaterials with high outputs yet a simple setup.

## Graphical abstract

Magnetically aligned ultrafine cobalt embedded 3D porous carbon metamaterial is synthesized by combining one‐step laser direct writing with external magnetic field. Superfast production speed of 2.5 cm^2^ s^−1^ and ultrafine Co nanoparticles (NPs) of 3.80 nm can be obtained massively. Significant improvements of electrocatalytic performances are found owning to magnetic field manipulation which leads to the well‐distributed G@Co NPs.

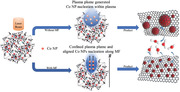

## 1 Introduction

Metamaterials are commonly described as structured materials with multiple periodically aligned building blocks which exhibit special properties that differ from and superior than the simply combined constituent materials.^[^ ^1^ ^]^ Besides constituent materials’ intrinsic properties, the internal, well‐designed structures play a critical role in their physical properties. Owing to this structure manipulation, recent studies have shown that metamaterials are capable of manipulating photonic,^[^ ^2^ ^]^ mechanical,^[^ ^1^, ^3^, ^4^ ^]^ acoustic,^[^ ^5^ ^]^ and thermal fields.^[^ ^6^ ^]^ These extraordinary metamaterials also bring to brand‐new interdisciplinary applications, such as electromagnetic wave absorbers^[^ ^7^ ^]^ and electro‐optical metadevices.^[^ ^8^ ^]^ The artificial structures of metamaterials are often made by micro/nanomanufacturing techniques (electron beam lithography,^[^ ^9^ ^]^ atomic layer deposition^[^ ^10^ ^]^) or 3D printing such as stereolithography.^[^ ^4^ ^]^ One of the major drawbacks to adopting these methods is time consuming and costly. Laser direct writing (LDW), however, provides a high‐throughput and flexible method to fabricate sensors,^[^ ^11^ ^]^ energy storage devices,^[^ ^12^ ^]^ catalyst,^[^ ^13^ ^]^ and mechanical metamaterials.^[^ ^14^ ^]^ Polymers and other carbonaceous materials are usually used as a precursor which would be further converted to carbons by laser. Previous studies show that 3D porous graphene material can be fabricated by one‐step laser scribing of polyimide films^[^ ^13^ ^]^ and woods.^[^ ^15^ ^]^ Versatile functionalities of the product could be further enhanced, by introducing metal salts into the polymer precursor to obtain metal/graphene or metal oxide/graphene composite materials after lasing. Ren et al.^[^ ^16^ ^]^ synthesized ternary metal oxide/graphene hybrid materials from lasing polyimide film and Co/Mn/Ni/Fe nitrides that are useful for both oxygen reduction reaction (ORR) and oxygen evolution reaction (OER) catalysis. Ultrafine Co_3_O_4_/graphene composite electrode material for high‐rate supercapacitors was also developed by Cao's team^[^ ^17^ ^]^ by KrF excimer laser converted Co_3_O_4_/graphene oxides. A key problem with much of the literature discussed above is the mixing of the precursor, which was usually carried out by drop casting or magnetic stirring. The discrete uniformity would alter the photothermal effects during laser treatment and thus causing defects in the final product. An atomic‐scale mixing should be a better candidate for solving this problem.

Metal–organic frameworks (MOFs), constructed by an atomic assembly of metal cations and oriented organic linkers,^[^ ^18^ ^]^ are ideal precursors for LDW to produce metamaterials. Previous works showed that MOFs could be converted to metal or metal oxide‐containing carbons by laser.^[^ ^19^, ^20^ ^]^ Different metal‐carbon composites were obtained by altering the metal cations in those MOFs. ^[^ ^20^, ^21^ ^]^ Unlike the traditional furnace annealing to convert ZIF‐67, a cobalt MOF, to the corresponding Co nanoparticles (NPs) decorated porous carbon, the cobalt nanoparticles were eager to coalesce under a prolonged heating process, thus large Co particles were obtained. In contrast, the size of the NPs was significantly reduced by using nanosecond (ns) laser radiation.^[^ ^20^ ^]^ However, the metal NPs converted by picosecond laser and the precise position manipulation of these MNPs as well as the carbon support are unexplored and challenging. It would be possible to manipulate the particles’ spatial arrangement under an external force field, such as magnetic field (MF).^[^ ^22^, ^23^ ^]^

## 2 Results and Discussion

Herein, we utilized picosecond (ps) laser as an energy source to directly convert ZIF‐67 crystals, a cobalt‐based MOF into well‐aligned ultrafine cobalt embedded 3D carbon metamaterials under the confinement of MF. Compared with ns or continuous laser, ps laser can produce transient nonequilibrium high temperature and pressure condition within few picoseconds,^[^ ^24^ ^]^ which precludes the coalescence of cobalt NPs. The rapid calcination of the organic component in MOF by ps laser leads to the formation of a 3D interconnected carbon framework by the thermal decomposition and subsequent laser welding. During the reduction of Co NPs, paramagnetic Co NPs were manipulated by the external magnetic field once they were nucleated to the critical size and aligned on the 3D carbons without further growth. Owning to the high heat and thermal transfer from MF‐induced laser plasma plume, its elemental composition is also refined. Electrochemical water splitting tests using this magnetic field‐aligned ultrafine Co embedded carbon as catalyst shows that this metamaterial exhibits a superior catalytic performance than that synthesized without MF, suggesting that spatial distribution of MNPs and controlled chemical states induced by MF is a practical way for the fabrication of metamaterials.

**Figure 1:**
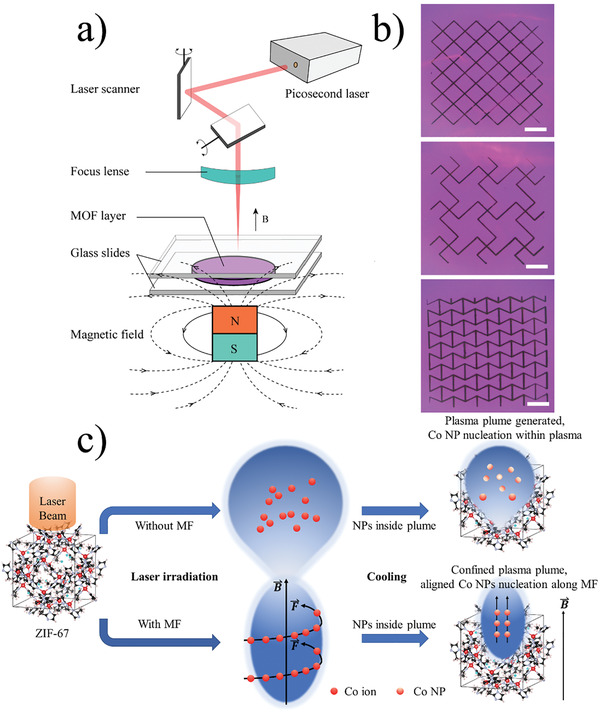
a) Experimental setup for MF‐assisted LDW. b) Several 2D metapatterns achieved by MF‐assisted ultrafast LDW: truss, chiral truss, and re‐entrant Honeycomb, the scale bar is 5 mm for all. c) Schematic illustration for the formation of magnetic aligned Co NPs. The paramagnetic ultrafine Co NPs are spatially aligned within the carbon matrix by MF.

The preparation of the magnetic field‐aligned ultrafine Co NPs embedded carbon is illustrated in **Figure** **1**a and the detailed experimental description is provided in Supporting Information. Typically, a ZIF‐67/n‐methyl‐2‐pyrrolidone (NMP) slurry was pasted onto a metal foil and dried at 110 °C for 12 h. This precursor preparation process could be facilely scaled up for mass production. A 100 µm ZIF‐67 coated copper foil was sandwiched by two glass slides and was placed upon a magnet. Afterward, the ZIF‐67 layer was scribed by a ps laser with precisely tuned pulse energy. Upon laser scribing, the purple ZIF‐67 layer instantly turned into black. The black product was further washed and dried, giving the final products named by ZIF67‐11M, ZIF67‐12M, and ZIF67‐13M (11, 12, and 13 represent the laser energy and M represents the magnetic field involved) and ZIF67‐11, ZIF67‐12, and ZIF67‐13 (lased by laser energy 11, 12, and 13 without MF). The whole process is ultrafast. For example, a 5 × 5 cm square sample area could be processed within 10 s, giving a high yield of 41.3%. Samples prepared by furnace annealing at 800 °C for 2 h were also synthesized by control and termed ZIF67‐a. Arbitrary patterns could be scribed by programmed laser writing, as shown in Figure 1b, providing the feasibility for mass production and smart design. Figure 1c illustrates the difference between Co NPs with or without MF involvement. When high power pulsed laser beam (5 ps, ≈100 GW cm^−2^) targets on material, laser ablation would occur and material is typically converted to a plasma plume with charged species (ions, clusters particulates, etc.). The process starts with the decomposition of ZIF‐67, as the coordination between Co ions and organic linkers (2‐methylimidazole, HMIM) from secondary building units in ZIF‐67 structures which allows the efficient absorption of light at a wavelength covering ultraviolet to near‐infrared. In the meantime, the intense laser beam shoots directly into the Co ions from ZIF‐67 and the carbonaceous organic linkers, HMIM are converted to reductive species followed by the reduction of Co ions to Co atoms within the plasma. The Co clusters further accumulate randomly and form Co NPs inside the 3D carbon framework, as stated in the upper flow in Figure 1c. The introduction of external MF (Surface field: 5754 Gauss), however, reshaped the pulse laser‐ZIF‐67 interactions (lower flow in Figure 1c. As the combination of plasma plume and magnetic force, charged species *q* within the plume (electric field **E**) would move with a velocity **v,** and the MF **B** experiences a force of ***F *** =  *q**E*** + *q**v*** × ***B***, the Lorentz force. This electromagnetic force constrains the plasma expansion in the vertical direction, as shown in Figure 1c ^[^ ^22^ ^]^ and the confined plasma plume decreases the plasma average distance to ZIF‐67 crystals. For laser–matter interactions without MF, the scattered mass tends to deposit at the center of the plasma plume. In contrast, the existence of MF breaks the large droplets into smaller ones and the deposited mass, Co NP is more uniform from the center to the edge.^[^ ^25^ ^]^ The MF would also induce electric current in the plasma which generates the Joule heating effect that leads to an increased plasma temperature and its thermal conductivity by solving the material hydrodynamic equation,^[^ ^26^ ^]^ with equations provided in the Supporting Information. Besides the MF‐induced laser–matter effects, MF also plays a critical role in the manipulation of Co NPs within the plasma during the cooling. Co clusters are aligned along with the MF once nucleated, and then take no time to accumulate, resulting in finer particle size and uniform distribution. The details of MF‐influenced NP size distribution would be discussed in the following chapters.

**Figure 2:**
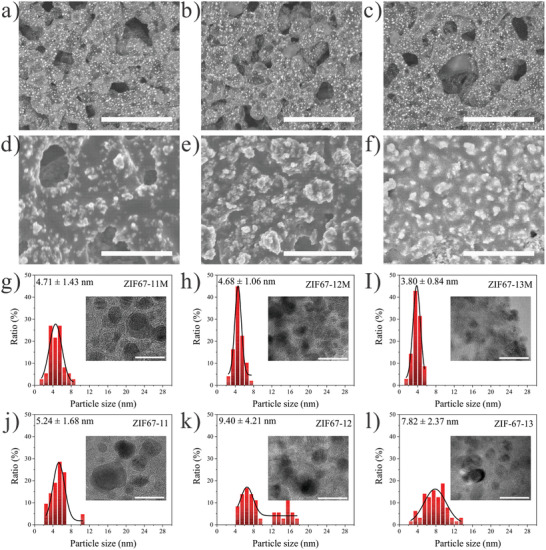
Backscattered electron SEM image of ps laser‐treated ZIF‐67 with or without magnetic field. a) ZIF67‐11M, b) ZIF67‐12M, and c) ZIF67‐13M and their corresponding d) ZIF67‐11, e) ZIF67‐12, and f) ZIF67‐13 without magnetic field. The scale bar is 5 µm for all. g–l) Size distributions of cobalt NPs within carbon matrix, summarized from TEM images. The scale bar is 20 nm for all inset TEM images.

The morphology of Co NPs was studied by scanning electron microscope (SEM). Backscattered electron image analysis was used to separate the signal of Co NPs from carbon species by the difference in atom numbers. A series of images is shown in **Figure** **2**a–c stands for samples with the assist of MF and d–f) for those without MF under the same lasing conditions. Dense, well‐distributed Co NPs and 3D interconnected carbon framework could be seen in MF‐assisted samples, while their counterparts have larger and random‐distributed Co NPs with chunk carbon supports. This significant contrast provides evidence that MF contributes to finer and well‐aligned Co NPs. Brunauer–Emmett–Teller surface area is carried out and the specific surface area for ZIF67‐13 is 247.95 m^2^ g^−1^ and ZIF67‐13M is 281.78 m^2^ g^−1^. The increased plasma plume by MF not only improves the product crystallinity but also induces more porosity in the carbon supports. We also studied the formation of a 3D porous carbon backbone. Figure S1 in the Supporting Information suggests that the stacking of pristine ZIF‐67 crystals helps the formation of 3D structures. Different amounts of ZIF‐67 slurries were dropped cast on Cu foil to investigate the impact of crystals stacking under the same lasing condition. Same laser conditions were carried out and the morphology of converted carbons are compared with pristine ZIF‐67. All samples were converted to “glued” carbons by laser; however, the carbons are only welded within their own stack when fewer MOF particles were added to form a loose stacking, as shown in Figure S1d,e in the Supporting Information. Interconnected 3D carbons with higher integrity only appear in compact stacking from Figure S1f in the Supporting Information, suggesting that the pristine ZIF‐67 stacking is important for the formation of macro 3D structure.

**Figure 3:**
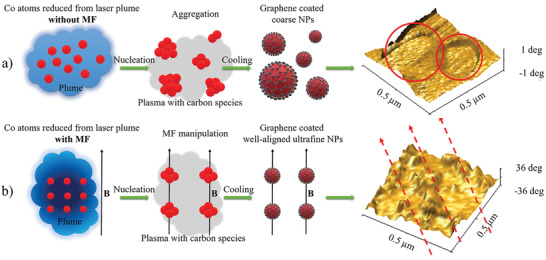
Schematic for Co NP formation: a) coarse and nonuniform Co NPs generated without MF manipulation and MFM image for ZIF67‐13 and b) ultrafine, dense, and aligned Co NPs generated with MF manipulation and MFM image for ZIF67‐13M.

Transmission electron microscope (TEM) is used to uncover the particle distribution on the 3D carbon backbone. We summarized the size distribution of Co NPs from high‐resolution TEM images and plotted them accordingly to find the difference (Figure 2g–l and Figures S2–S7, Supporting Information). Figure 2g–i are those from MF‐assisted products, ZIF67‐11M (4.71 ± 1.43 nm), ZIF67‐12M (4.68 ± 1.06 nm), and ZIF67‐13M (3.80 ± 0.84 nm) and Figure 2j–l are their counterparts, ZIF67‐11 (5.24 ± 1.68 nm), ZIF67‐12 (9.40 ± 4.21 nm), and ZIF67‐13 (7.82 ± 2.37 nm), respectively. From samples prepared without MF assistance, higher laser energy results in larger particle size and wider distribution. This should be caused by higher heating effects from lasing which facilities the Co NP growth. As discussed in **Figure** **3**a, the Co atoms are first reduced from Co ions during laser–matter interactions. Then the Co atoms start to grow as Co clusters formed. Aggregation and coalescence of the NPs happen as follows and Co NPs with random sizes are thus derived, as demonstrated in the atomic force microscopy (AFM) image. On the contrary, all three samples with MF have a narrowed size distribution and smaller particle size (≈4 nm). Additional AFM images also match well with the SEM and TEM results (Figure S8, Supporting Information). The surface patterns of ZIF‐67‐13M are well‐aligned arrays with smaller particles other than the coarse surface in ZIF‐67‐13. Previous works studied the size effects in the magnetic anisotropy of embedded cobalt nanoparticles and stated that clusters with diameters >3 nm are critical.^[^ ^27^ ^]^ Here we propose that during our MF‐assisted LDW process, the Co NPs once reached 3 nm, would be directly dragged by the Lorentz force and anchored on the carbon backbone, precluding the particle aggregation issue and lead to a finer and dense NP distribution (Figure 3b). The magnetic force microscopy (MFM) images in Figure 3a show two Co domains from ZIF67‐13. The one with a positive degree is dragged and the other with a negative degree is pulled by the MFM tip. Stronger magnetic force and uniform Co arrays could be observed within ZIF67‐13M (Figure S8e,f, Supporting Information). In conclusion, our SEM, TEM, and AFM data crossvalidate that the MF plays a vital role in refining and aligning the Co NPs within the carbon backbone.

**Figure 4:**
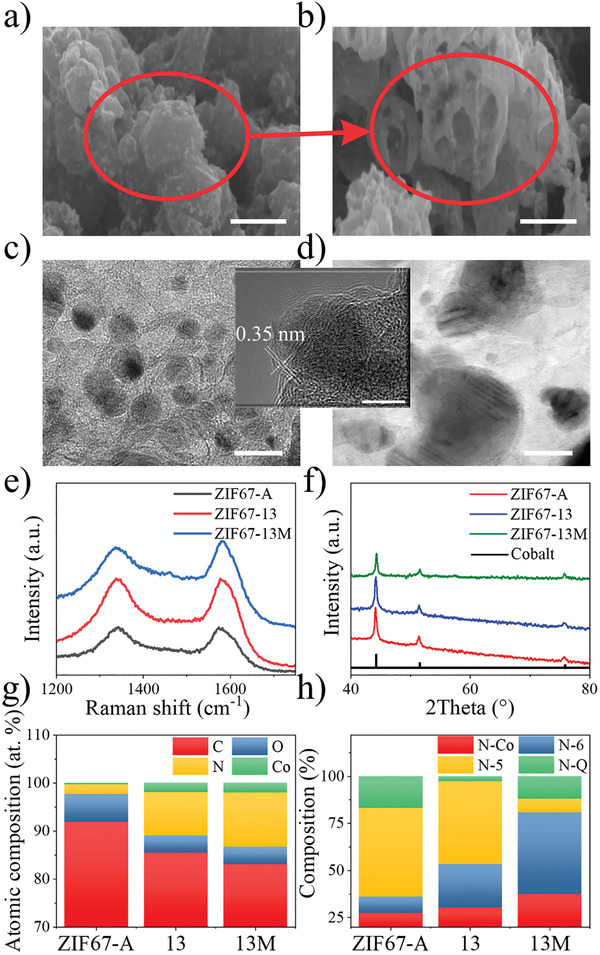
Characterization of ZIF67 samples. SEM images of a) ZIF67‐13M and b) the exposed inner parts by FIB cutting. The scale bar is 2 µm for (a) and 1 µm for (b). TEM images of c) ZIF67‐13M and d) ZIF67‐13. The scale bar is 20 nm for each. The inset TEM image between (c) and (d) shows the graphene layer, with a scale bar of 5 nm. e) Raman and f) XRD for ZIF67 samples. g) Atomic composition and h) XPS N1s composition derived from XPS data.

ZIF‐67‐13M was selected to better understand their physical properties. As shown in Figures S2–S7 in the Supporting Information, all samples have a 3D interconnected carbon framework on a macroscale. Interestingly, the inner side of the carbon backbone is still porous, evidenced by a broken piece of carbon in Figure S4e in the Supporting Information. Herein, an intact carbon backbone of ZIF67‐13M was selected and was cut by a focused ion beam (FIB) to examine the inner porosity (**Figure** **4**a). After FIB cutting, the inner section was exposed and the sponge‐like interconnected structure was observed (Figure 4b). Elemental mapping was also performed by TEM energy dispersive spectrometer to illustrate the ZIF‐67‐13M's elemental composition (Figure S9, Supporting Information). The N element, derived from HMIM, distributes evenly across the whole carbon surface while the particles are verified to be cobalt. Overall, our magnetically aligned ultrafine cobalt NPs embedded in 3D porous carbon metamaterial exhibits predesigned hierarchical 3D structure, from porosity in microscale to macroscale. Subsequently, the TEM image in Figure 4c demonstrates the uniform distribution of Co NPs in ZIF67‐13M and the coarse Co NPs could be seen for ZIF67‐13 in Figure 4d. Notably, the carbon surrounding each Co particle is few‐layered graphene instead of the backbone's amorphous state (inset TEM images between (c) and (d)). Recent research discussed the growth of graphene by cobalt‐catalyzed decomposition of hydrocarbon through the chemical vapor deposition (CVD) process.^[^ ^28^ ^]^ We suppose that the carbonaceous species decomposed from HMIM not only reduce Co ions but also solute in the converted Co particles (≈1 atom % at 1000 °C) during the laser heating stage. Followed by the ultrafast cooling enabled by ps laser, the carbon segregates on the Co surface, forming a few‐layers of graphene as a protection layer of Co NPs.

Raman spectrum from Figure 4e further supports the existence of graphene. The peaks observed at 1349 and 1590 cm^−1^ are the D (defects and disorder) and G (graphitic) bands of carbon materials, respectively.^[^ ^29^ ^]^ Among all samples, ZIF67‐13M has the highest G/D ratio (1.05 over 0.98 for ZIF67‐13; 0.95 for ZIF67‐A), indicating a better graphene crystallinity which could also be found in TEM images. This could be explained by the higher plasma temperature induced by MF during laser irradiation. X‐ray powder diffraction (XRD) was tested in Figure 4f to confirm the reduction of Co ion. Two significant peaks could be found at 2*θ* = 44.2° and 51.5°, which are attributed to the (100) and (200) crystal planes of the metallic Co (JCPDS No. 15‐0806), respectively.^[^ ^30^ ^]^ All samples have strong signals of Co and no signals from cobalt oxides could be found. Elemental compositions of ZIF67 samples from X‐ray photoelectron spectroscopy (XPS) spectra are illustrated in Figure 4g and the N1s spectra of ZIF67‐13M display four peaks centered at 398.5, 399.5, 400.9, and 402.4, corresponding to N‐Co, N‐6, N‐5, and N‐Q, respectively^[^ ^31^, ^32^ ^]^ (Figure S10, Supporting Information). The N ratios from MF‐assisted LDW samples are higher than that from traditional furnace annealing, where the N‐doped carbon has also been proved to strengthen the electrocatalysis performance.^[^ ^33^ ^]^ The laser processed ZIF‐67 has ≈2 at% of Co while only 0.28 at% of Co could be seen in ZIF67‐A. This is probably due to the sufficient reduction by laser irradiation while cobalt oxides might be formed in ZIF67‐A during annealing and then be washed away by HCl. ZIF67‐13M has the highest N ratio of 11.34 at% and followed by 9.06 at% for ZIF67‐13 and 2.03 at% for ZIF67‐A. The N1s composition in Figure 4h states the importance of LDW process. N‐Q is 16.81, 2.72, and 11.92 at% for ZIF67‐A, ZIF67‐13, and ZIF67‐13M, respectively. The N‐Q has been widely reported to play important roles in catalytic activity and is usually formed in high‐temperature and high‐pressure conditions. ^[^ ^32^, ^34^ ^]^ It has also been suggested that the atomic magnetization of the reaction center might serve as a descriptor of the catalytic activity,^[^ ^35^ ^]^ and the electron spin polarization of Co on the microlevel could enhance catalytic activities.^[^ ^36^ ^]^ Given these points, the one‐step LDW process enables an ultrafast and high‐yield synthesis process of well‐aligned ultrafine cobalt embedded 3D carbon metamaterials, which would be a suitable candidate as an electrocatalyst for water splitting.

**Figure 5:**
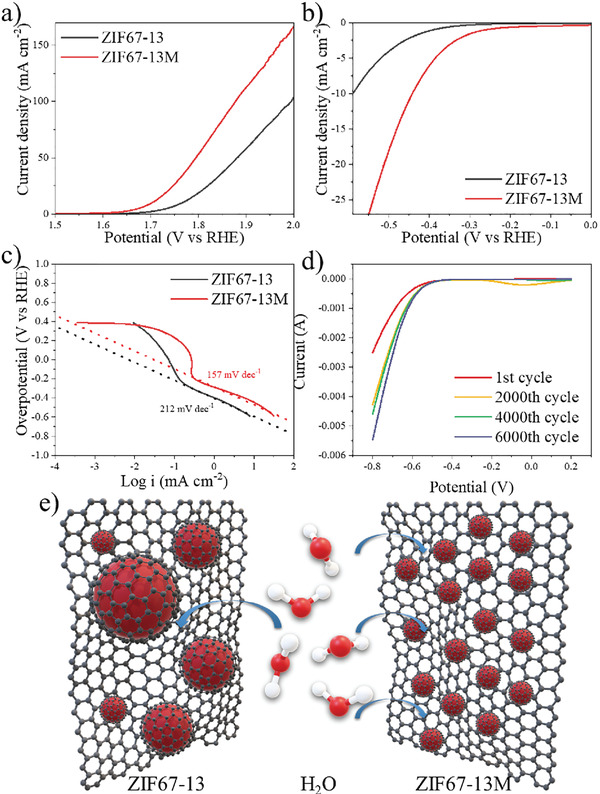
Electrocatalytic performance of ZIF67‐13M. a) OER polarization curves for ZIF67‐13 and ‐13M under 50 mV s^−1^. b) HER polarization curves for ZIF67‐13 and ‐13M under 50 mV s^−1^ and c) corresponding Tafel slopes. d) HER polarization curves before and after 6000 potential sweeps. e) Schematic for ZIF67‐13/13M's electrocatalytic behavior underwater splitting.

**Figure 6:**
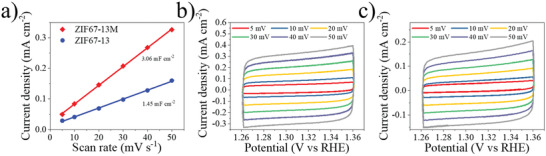
a) The difference in current density plotted against scan rate of ZIF67‐13M and ZIF67‐13 for estimating the double layer capacitances and corresponding CV curves for b) ZIF67‐13M and c) ZIF67‐13 at different scan rates (5, 10, 20, 30, 40, and 50 mV s^−1^).

The electrocatalytic activities of ZIF67‐13 and ZIF67‐13M for OER and hydrogen evolution reaction (HER) were evaluated using a typical three‐electrode system. The catalytic performance of OER was assessed in 1 m NaOH solution and HER was in 0.5 m H_2_SO_4_ solution. Polarization curves were obtained from linear sweep voltammetry (LSV) measurements with a sweep rate of 50 mV s^−1^ (**Figure** **5**a). ZIF‐67‐13M shows an overpotential of ≈0.45 V at current density of 10 mA cm^−2^, which is remarkably ≈90 mV lower than that of ZIF‐67‐13 (≈0.54 V) at the same current density. OER LSV for all samples are demonstrated in Figure S11 in the Supporting Information and overpotential differences without or with MF during LDW are 80, 20, 20 mV, and 30, 20, 90 mV under 10 and 50 mV s^−1^ for 11, 12, and 13 laser conditions, respectively (Tables S1 and S2, Supporting Information). All samples under MF alignments show significant improvements over their counterparts (for example, 14.3% improvement for 13 m over 13), giving evidence that the metastructure formation by MF manipulation is crucial for the catalysis process. Meanwhile, the abundant N‐Q in ZIF67‐13M also contributes to the improvement, as it shows the highest active sites. Electrochemically active surface area (ECSA) is another important parameter to reflect the number of active sites on the surface of the catalysis during electrochemical reactions.^[^ ^37^ ^]^ The ECSA could be estimated by the electrochemical double‐layer capacitance (*C*_dl_), which is calculated from the cyclic voltammetry (CV) curves performed in a nonfaradic region (**Figure** **6**). The numerical value of the ECSA is corresponding to the double‐layer capacitance. In Figure 6a, ZIF67‐13M has a *C*_dl_ value of 3.06 mF cm^−2^, which is 2.11 times higher than that of ZIF67‐13 (1.45 mF cm^−2^). This indicates that ZIF67‐13M possesses more exposed active sites than ZIF67‐13 and is intrinsically more active, as the result of the unique MF‐assisted laser irradiation process.

HER testing was further carried out to validate the importance of MF manipulation. Figure 5b shows the LSV curves and corresponding Tafel slopes of ZIF67‐13 and ‐13M with a sweep rate of 50 mV s^−1^. The potential of ZIF67‐13M is 150 mV greater than that of ZIF67‐13 (−0.59 mV) to reach the current density of 10 mA cm^−2^. As shown in Figure 5c, ZIF67‐13M has a Tafel slope of 157 mV dec^−1^, which is also smaller than that of ZIF67‐13 (212 mV dec^−1^). Figure 5d demonstrates that the HER LSV curves of ZIF67‐13M after 6000 cycles. Interestingly, the ZIF67‐13M was activated during the cycling and the overpotentials were gradually lowered down. As mentioned before, few layers of graphene formed on top of the Co NPs by the possible CVD mechanism during the lasing in ambient conditions. Our catalytic durability test suggests the importance of excellent chemical protection enabled by the coated graphene layers where it is barely seen by other fabrication methods. The physical durability was also examined by magnetic absorption in Figure S12 in the Supporting Information. Both ZIF‐67‐13 and ‐13M still maintained their paramagnetic properties even after one year while the ZIF67‐A, made by traditional furnace annealing, was oxidized, as barely no absorption could be observed. All these results point to the critical role of MF manipulations during LDW, as illustrated in Figure 5e. Unlike ZIF67‐13, ZIF67‐13M's Co NPs are smaller and more uniform. Given by the precursor materials, ZIF‐67 crystal with a fixed ratio of Co ions over organic linkers, the smaller particle sizes would generate more Co particles. As reported by Feng and co‐workers,^[^ ^38^ ^]^ the well‐distributed active sites from catalyst would benefit the electrocatalytic activities. Our experimental data proved that the MF‐alignment of Co NPs could form uniform and abundant active sites (N‐Q) along with the 3D interconnected carbons and providing unexpected electrocatalytic performances than those samples without MF regulation.

## 3 Conclusion

In conclusion, we for the first time introduced MF into the LDW process to directly manipulate the spatial distribution of the generated paramagnetic NPs. A piece of 5 × 5 cm^2^ of ultrafine cobalt embedded 3D porous carbon metamaterial could be synthesized within 10 s with one‐step laser scribing in air. Uniform graphene layers were also formed on the surface of each Co NPs during the laser processing, providing an excellent protection layer for Co NPs. The hierarchical carbonaceous structure was also obtained during the conversion of MOF by MF confinement laser scribing with abundant N‐Q species. Our OER and HER studies proved the importance of MF, as overpotential differences of 90 mV for OER and 150 mV for HER to reach 10 mA cm^−2^ than those without MF. Excellent cycling performance up to 6000 cycles without decay was also obtained owning to the graphene coating. Both NP size and distribution together with its elemental composition are well‐designed by a simple one‐step MF‐assisted LDW process. Our multifield collaborative method opens a new path for designing metamaterials by combining photothermal field and external electromagnetic field to form unique physical and chemical properties, leading to their potential applications for electrochemical energy storage, microwave absorption, magnetothermal effects, and beyond.

## Conflict of Interest

The authors declare no conflict of interest.

## Supporting information

Supporting Information

## Data Availability

Research data are not shared.

## References

[1] K. Bertoldi , V. Vitelli , J. Christensen , M. van Hecke , Nat. Rev. Mater. 2017, 2, 17066.

[2] a) C. M. Soukoulis , M. Wegener , Nat. Photonics 2011, 5, 523;

[3] X. Xu , Q. Zhang , M. Hao , Y. Hu , Z. Lin , L. Peng , T. Wang , X. Ren , C. Wang , Z. Zhao , C. Wan , H. Fei , L. Wang , J. Zhu , H. Sun , W. Chen , T. Du , B. Deng , G. J. Cheng , I. Shakir , C. Dames , T. S. Fisher , X. Zhang , H. Li , Y. Huang , X. Duan , Science 2019, 363, 723. 30765563 10.1126/science.aav7304

[4] B. Deng , R. Xu , K. Zhao , Y. Lu , S. Ganguli , G. J. Cheng , Mater. Today 2018, 21, 467.

[5] a) X. F. Zhu , K. Li , P. Zhang , J. Zhu , J. T. Zhang , C. Tian , S. C. Liu , Nat. Commun. 2016, 7, 7; 10.1038/ncomms11731 PMC4876457 27198887

[6] Q. M. Wang , J. A. Jackson , Q. Ge , J. B. Hopkins , C. M. Spadaccini , N. X. Fang , Phys. Rev. Lett. 2016, 117, 6. 10.1103/PhysRevLett.117.175901 27824463

[7] C. M. Watts , X. Liu , W. J. Padilla , Adv. Mater. 2012, 24, OP98. 22627995 10.1002/adma.201200674

[8] H.‐T. Chen , W. J. Padilla , J. M. O. Zide , A. C. Gossard , A. J. Taylor , R. D. Averitt , Nature 2006, 444, 597. 17136089 10.1038/nature05343

[9] N. Liu , H. Guo , L. Fu , S. Kaiser , H. Schweizer , H. Giessen , Nat. Mater. 2008, 7, 31. 18059275 10.1038/nmat2072

[10] Q. Zhang , D. Lin , B. Deng , X. Xu , Q. Nian , S. Jin , K. D. Leedy , H. Li , G. J. Cheng , Adv. Mater. 2017, 29, 1605506. 10.1002/adma.201605506 28556473

[11] Y. Gao , Q. Li , R. Wu , J. Sha , Y. Lu , F. Xuan , Adv. Funct. Mater. 2019, 29, 1806786.

[12] J. Yi , J. Chen , Z. Yang , Y. Dai , W. Li , J. Cui , F. Ciucci , Z. Lu , C. Yang , Adv. Energy Mater. 2019, 9, 1901796;

[13] Z. Jibo , R. Muqing , W. Luqing , L. Yilun , B. I. Yakobson , J. M. Tour , Adv. Mater. 2018, 30, e1707319.

[14] T. Bückmann , N. Stenger , M. Kadic , J. Kaschke , A. Frölich , T. Kennerknecht , C. Eberl , M. Thiel , M. Wegener , Adv. Mater. 2012, 24, 2710. 22495906 10.1002/adma.201200584

[15] R. Ye , Y. Chyan , J. Zhang , Y. Li , X. Han , C. Kittrell , J. M. Tour , Adv. Mater. 2017, 29, 1702211. 10.1002/adma.201702211 28737226

[16] M. Ren , J. Zhang , J. M. Tour , ACS Appl. Energy Mater. 2019, 2, 1460.

[17] S. Yang , Y. Liu , Y. Hao , X. Yang , W. A. Goddard , X. L. Zhang , B. Cao , Adv. Sci. 2018, 5, 1700659. 10.1002/advs.201700659 PMC5908357 29721414

[18] S. Yuan , L. Feng , K. Wang , J. Pang , M. Bosch , C. Lollar , Y. Sun , J. Qin , X. Yang , P. Zhang , Q. Wang , L. Zou , Y. Zhang , L. Zhang , Y. Fang , J. Li , H.‐C. Zhou , Adv. Mater. 2018, 30, e1704303. 10.1002/adma.201704303 29430732

[19] A. Basu , K. Roy , N. Sharma , S. Nandi , R. Vaidhyanathan , S. Rane , C. Rode , S. Ogale , ACS Appl. Mater. Interfaces 2016, 8, 31841. 27809473 10.1021/acsami.6b10193

[20] H. Jiang , S. Jin , C. Wang , R. Ma , Y. Song , M. Gao , X. Liu , A. Shen , G. J. Cheng , H. Deng , J. Am. Chem. Soc. 2019. 10.1021/jacs.9b00355 30823704

[21] H. Jiang , L. Tong , H. Liu , J. Xu , S. Jin , C. Wang , X. Hu , L. Ye , H. Deng , G. J. Cheng , Matter 2020, 2, 1535.

[22] a) C. Ye , G. J. Cheng , S. Tao , B. Wu , J. Manuf. Sci. Eng. 2013, 135;

[23] a) X. Yu , J. Yu , Y. Fautrelle , A. Gagnoud , Z. Ren , X. Lu , X. Li , J. Mater. Chem. A 2019, 7, 19733;

[24] G. Qiu , Q. Nian , M. Motlag , S. Jin , B. Deng , Y. Deng , A. R. Charnas , P. D. Ye , G. J. Cheng , Adv. Mater. 2018, 30, 1704405. 10.1002/adma.201704405 29337377

[25] K. S. Singh , A. K. Sharma , J. Appl. Phys. 2016, 119, 183301.

[26] B. Wu , S. Tao , Y. Gao , Y. Zhou , G. Cheng presented at ASME 2011 Int. Mech. Eng. Congr. Expo., Denver, Colorado, USA 2011.

[27] S. Oyarzún , A. Tamion , F. Tournus , V. Dupuis , M. Hillenkamp , Sci. Rep. 2015, 5, 14749. 26439626 10.1038/srep14749 PMC4593963

[28] a) E. Kim , H. An , H. Jang , W.‐J. Cho , N. Lee , W.‐G. Lee , J. Jung , Chem. Vap. Deposition 2011, 17, 9;

[29] J. Xu , Z. Tan , W. Zeng , G. Chen , S. Wu , Y. Zhao , K. Ni , Z. Tao , M. Ikram , H. Ji , Y. Zhu , Adv. Mater. 2016, 28, 5222. 27143563 10.1002/adma.201600586

[30] H. Guo , Q. Feng , J. Zhu , J. Xu , Q. Li , S. Liu , K. Xu , C. Zhang , T. Liu , J. Mater. Chem. A 2019, 7, 3664.

[31] I. S. Amiinu , X. Liu , Z. Pu , W. Li , Q. Li , J. Zhang , H. Tang , H. Zhang , S. Mu , Adv. Funct. Mater. 2018, 28, 1704638.

[32] Z. Guo , F. Wang , Y. Xia , J. Li , A. G. Tamirat , Y. Liu , L. Wang , Y. Wang , Y. Xia , J. Mater. Chem. A 2018, 6, 1443.

[33] a) C. Hu , L. Dai , Adv. Mater. 2019, 31, 1804672; 10.1002/adma.201804672 30566275

[34] a) Z. Zou , M. Cai , X. Zhao , J. Huang , J. Du , C. Xu , J. Mater. Chem. A 2019, 7, 14011;

[35] Y. Sun , J. Wang , Q. Liu , M. Xia , Y. Tang , F. Gao , Y. Hou , J. Tse , Y. Zhao , J. Mater. Chem. A 2019, 7, 27175.

[36] J. Yan , Y. Wang , Y. Zhang , S. Xia , J. Yu , B. Ding , Adv. Mater. 2021, 33, 2007525. 10.1002/adma.202007525 33336466

[37] a) C. Huang , T. Ouyang , Y. Zou , N. Li , Z.‐Q. Liu , J. Mater. Chem. A 2018, 6, 7420;

[38] Y. Hou , M. Qiu , M. G. Kim , P. Liu , G. Nam , T. Zhang , X. Zhuang , B. Yang , J. Cho , M. Chen , C. Yuan , L. Lei , X. Feng , Nat. Commun. 2019, 10, 1392. 30918251 10.1038/s41467-019-09394-5 PMC6437202

