# Supplementary material for: Magnetically Aligned Ultrafine Cobalt Embedded 3D Porous Carbon Metamaterial by One‐Step Ultrafast Laser Direct Writing
Source: Adv Sci (Weinh). 2021 Nov 1;8(24):2102477. doi: 10.1002/advs.202102477 (PMC8693064; doi:10.1002/advs.202102477)
Supplement: Supplementary file 1 — Supporting Information [file ADVS-8-2102477-s001.pdf]

© 2021 Wiley-VCH GmbH

ADVANCED SCIENCE
Open Access

Supporting Information

for *Adv. Sci.*, DOI: 10.1002/advs.202102477

Magnetically Aligned Ultrafine Cobalt Embedded 3D Porous Carbon Metamaterial by One-step Ultrafast Laser Direct Writing

*Jin Xu, Ruoxing Wang, Haoqing Jiang, Xingtao Liu, Licong An, Shengyu Jin, Biwei Deng, Wenzhuo Wu, Gary J. Cheng**

Supporting Information

Magnetically Aligned Ultrafine Cobalt Embedded 3D Porous Carbon Metamaterial by One-step Ultrafast Laser Direct Writing

*Jin Xu, Ruoxing Wang, Haoqing Jiang, Xingtao Liu, Licong An, Shengyu Jin, Biwei Deng, Wenzhuo Wu, Gary J. Cheng**

1. Experimental Section

Synthesis of ZIF-67: 5.82 g (0.02 mol) $Co(NO_3)_2{\cdot}6H_2O$ (Sigma Aldrich, NO. 203106) and 6.16 g (0.075 mol) 2-methylimidazole (Sigma Aldrich, NO. M50850) were dissolved in 75 ml methanol (Sigma Aldrich, NO. 34860) under magnetic stirring for 5 min to form clear solutions, respectively. Then the 2-methylimidazole solution was poured into $Co(NO_3)_2{\cdot}6H_2O$ solution under magnetic stirring for 5 min. The above mixed solution was aged at room temperature for 24 h. The purple precipitates were collected by centrifuging at 3000 rpm. The final ZIF-67 was obtained by washing with methanol for 3 times and dried at 60 °C for 12 h.

Sample preparation: ZIF-67 slurry was formed in N-methyl-2-pyrrolidone (NMP, Sigma Aldrich, NO. 328634) by sonication. The slurry obtained was pasted onto substrate (100 µm stainless steel foil (MTI Corp., NO. SSF-316-300-0) or 9 µm Cooper foil (MTI Corp., NO. BCCF-9U)) by using a doctor-blade to achieve a thickness of about 100 µm. The coated substrate was dried at 110 °C for 12h before use.

Laser system setup: A picosecond pulse fiber laser (Fianium HE-1060) was used as laser source. The wavelength was 1064 nm and the pulse width was 5 ps with the waveforms digitally controlled by computer. The laser spot size was focused to ca. 50 µm for the scanning processing by galvo mirrors. The writing speed of the laser is fixed to 10 mm s^{-1}.

Fabrication of magnetic aligned ultrafine Cobalt embeded 3D carbon meta-materials: The ZIF-67 coated SSF was sandwiched by two glass slides (Thermo Scientific, NO. 12-518-100B) and was placed with a magnet (Dimensions: 1" x 1" x 1" thick, Surface Field: 5754 Gauss, K&J Magnetics, Inc., NO. BX0X0X0,) beneath. After laser processing, the sample was taken out and washed with 1.0 M HCl (Sigma Aldrich, NO. 320331), DI water and ethanol (Sigma Aldrich, NO. 459836), which removed untreated ZIF-67. Various laser energies were applied with/without MF involvement, and the corresponding products are listed below:

**Laser energy**	**11**	**12**	**13**
**With MF**	ZIF67-11M	ZIF67-12M	ZIF67-13M
**Without MF**	ZIF67-11	ZIF67-12	ZIF67-13

Characterizations: The morphology of samples was characterized by scanning electron microscopy (SEM, Hitachi S-4800 Field Emission SEM), high-resolution transmission electron microscopy (HRTEM, FEI Talos 200X). The structure of samples was measured by X-ray diffraction spectroscopy (XRD, Panalytical Empyrean Powder X-ray diffractometer with both parallel beam and focusing optics and a high speed PIXcel 3D Medipix detector), and Raman spectroscopy (Horiba LabRAM HR, 532 nm laser). The composition of samples was measured by X-ray photoelectron spectroscopy (XPS, Kratos AXIS Ultra DLD). The surface area and pore size distribution of samples were calculated with N2 adsorption-

desorption using a Quantachrome instrument (autosorb iQ2) with Brunauer-Emmett-Teller (BET) methods and quenched solid density functional theory (QSDFT) model.

Electrochemical testing: The electrochemical performance of the samples was evaluated with CHI 660E equipped with a glassy carbon electrode (Dia: 3mm). A platinum wire serves as a counter electrode, Ag/AgCl electrode as the reference electrode and the samples as working electrodes. The electrolyte was 1 M NaOH and 0.5 M H_2SO_4. All potentials were converted to reversible hydrogen electrode (RHE) following the Nernst equation:

$$E_{RHE}(V) = E_{Ag/AgCl} + 0.235 + 0.059 \times pH$$

HER and OER polarization curves were recorded at the scan rate of 10 and 50 mV s^{-1}. The working electrodes were cycled at 10 mV s^{-1} until a stable cyclic voltammetry (CV) was achieved before we collected the data. Electrochemical capacitance was measured using CV measurements. The currents were measured in a narrow potential window that no faradaic processes were observed. CVs were collected at different scan rates: 5, 10, 20, 30, 40, and 50 mV s^{-1}. The measured current in this non-Faradaic potential region should be mostly due to the charging of the double-layer. By plotting the capacitive currents against the scan rate and following with a linear fit, the double layer capacitance C_{dl} is around half of the slope.

2. Statistical Analysis:

Co NP size distribution: HRTEM images (Figure S2-7) of ZIF67 samples are imported into the ImageJ to measure particle sizes. Sample size (n) is shown below:

**Sample**	**11**	**11M**	**12**	**12M**	**13**	**13M**
n	21	37	36	51	64	36

Measured particle sizes are grouped and separated by 1 nm to plot the pore size distribution by Origin 2020 in stacked column graph (0-1 nm, 1-2 nm,...,9-10 nm). The average size is calculated by the sum of the set of numbers divided by the count which is the number of the values being added. The standard deviation is calculated as the square root of variance by determining each data point's deviation relative to the mean. The curve ontop of the column graph is derived by the nonlinear curve fit (Gaussian) by Origin 2020.

XPS peak analysis: the atomic composition are collected directly from XPS data. The N1s composition is processed by XPSPEAK 4.1 peak optimization.

3. Theoretical Ananlysus of MF-induced plasma plume change:

In this paper, a hypothesized explanation is provided based on theoretical analysis:[1]

The high intensity laser beam would generate a plasma plume at the surface of ZIF-67 crystals, which would expand. When the external MF exists, it will induce electric current and hence electromagnetic force in the plasma, and the current density **J** and EM force **F** are given by: (1) and (2)

$$\boldsymbol{J} = \sigma(-\nabla\varphi + \boldsymbol{V} \times \boldsymbol{B})$$

$$\boldsymbol{F} = \boldsymbol{J} \times \boldsymbol{B}$$

Where σ is the plasma electric conductivity, φ is the electrostatic potential, $\boldsymbol{V}$ is the material velocity in plasma, and $\boldsymbol{B}$ is the MF. If the term of $\nabla\varphi$ is neglected (for the simplicity of analysis), then the current density can be given by: (3)

$$\boldsymbol{J} = \sigma\boldsymbol{V} \times \boldsymbol{B})$$

Fig. 1c) shows a schematic for the effect of magnetic field on plasma induced by laser irradiation, where the MF is assumed to be in +Z direction. Equations (2) and (3) imply that the motion of plasma material

in any direction (except the ±Z direction) would induce electric current and thus electromagnetic force that would constrain the motion. Hence, the electromagnetic force would confine the expansion of the plasma in any direction that is not parallel to the Z-axis. For example, it will confine the plasma expansion in ±X as shown in the lower part of Fig. 1c).

The plasma might absorb a huge amount of energy from the laser beam, and it might also transfer part of its energy to the workpiece through thermal conduction and other mechanism. A very rough estimation of the heat conduction flux, q, from the plasma to the ZIF-67 surface could be given by: (4)

$$q = k_e \frac{T_p - T_w}{L}$$

where k_e is the average plasma thermal conductivity, T_p is the plasma average temperature, T_w is the workpiece surface temperature, and L is the average distance from plasma to the workpiece surface in X direction.

The confinement of the plasma expansion in +X direction due to the electromagnetic force induced by the MF would decrease the value of L. The confinement effect would convert part of the plasma kinetic energy to its internal energy, and hence may increase the plasma temperature T_p. A higher plasma temperature would also increase the plasma thermal conductivity k_e. All of these would increase the value of the heat conduction flux from the plasma to ZIF-67, which could be seen from Eq. (4).

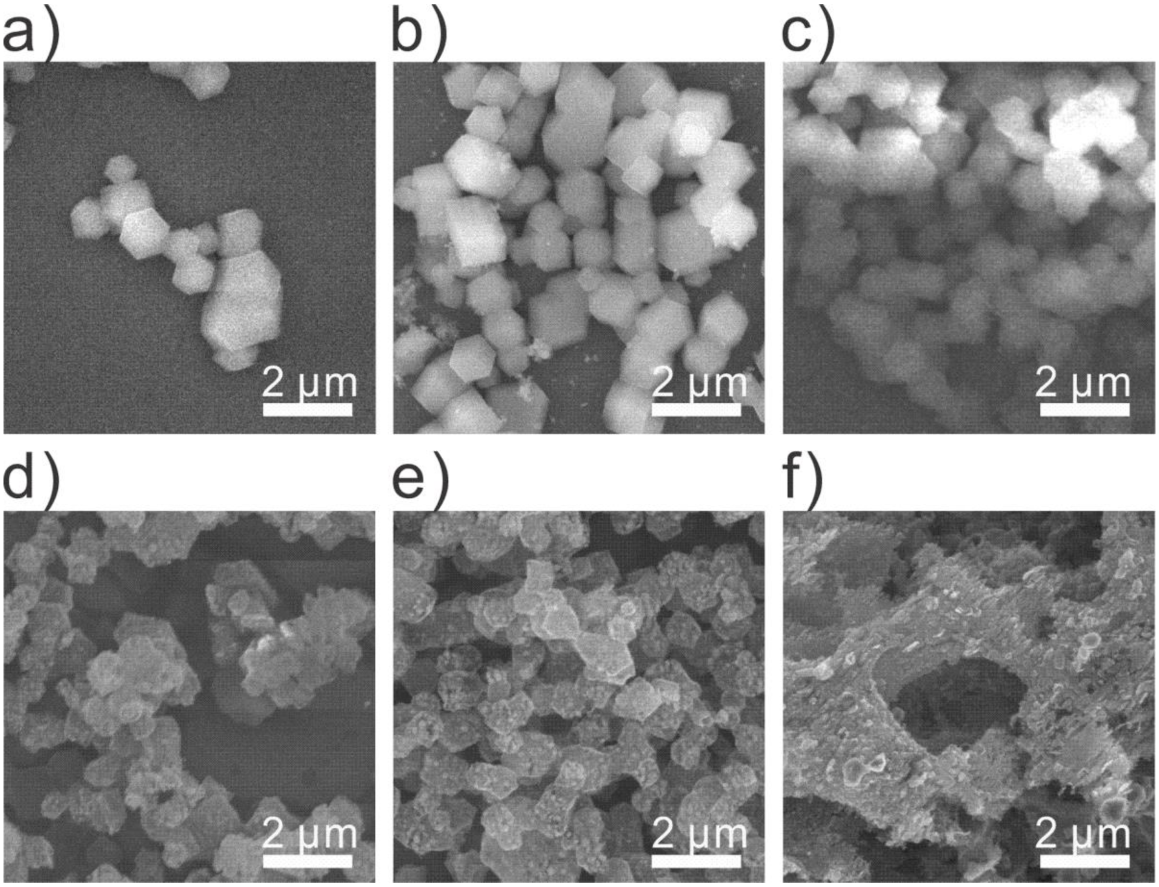

Figure S1. 3D porous carbon evolution: a), b), c) are pristine ZIF-67 crystals with increased stacking; d), e), f) are corresponding ps laser converted 3D carbon framework.

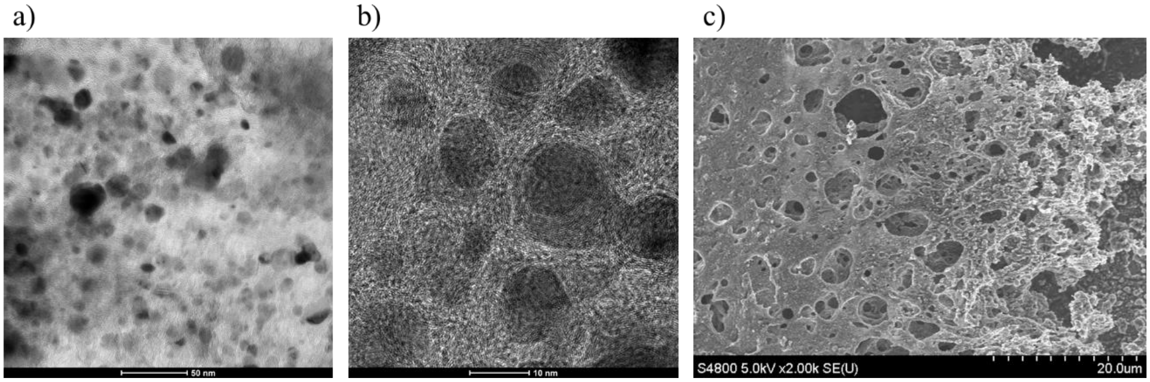

Figure S2. a, b) TEM and c) SEM images of ZIF67-11M.

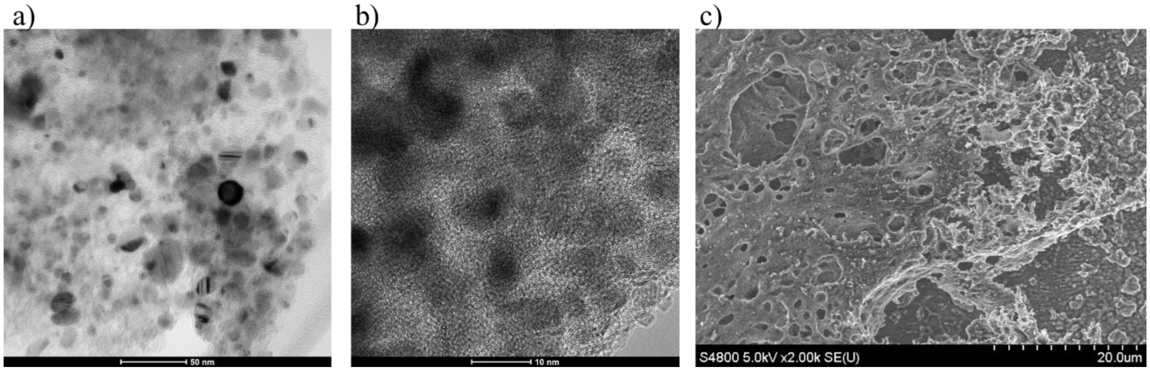

Figure S3. a, b) TEM and c) SEM images of ZIF67-12M.

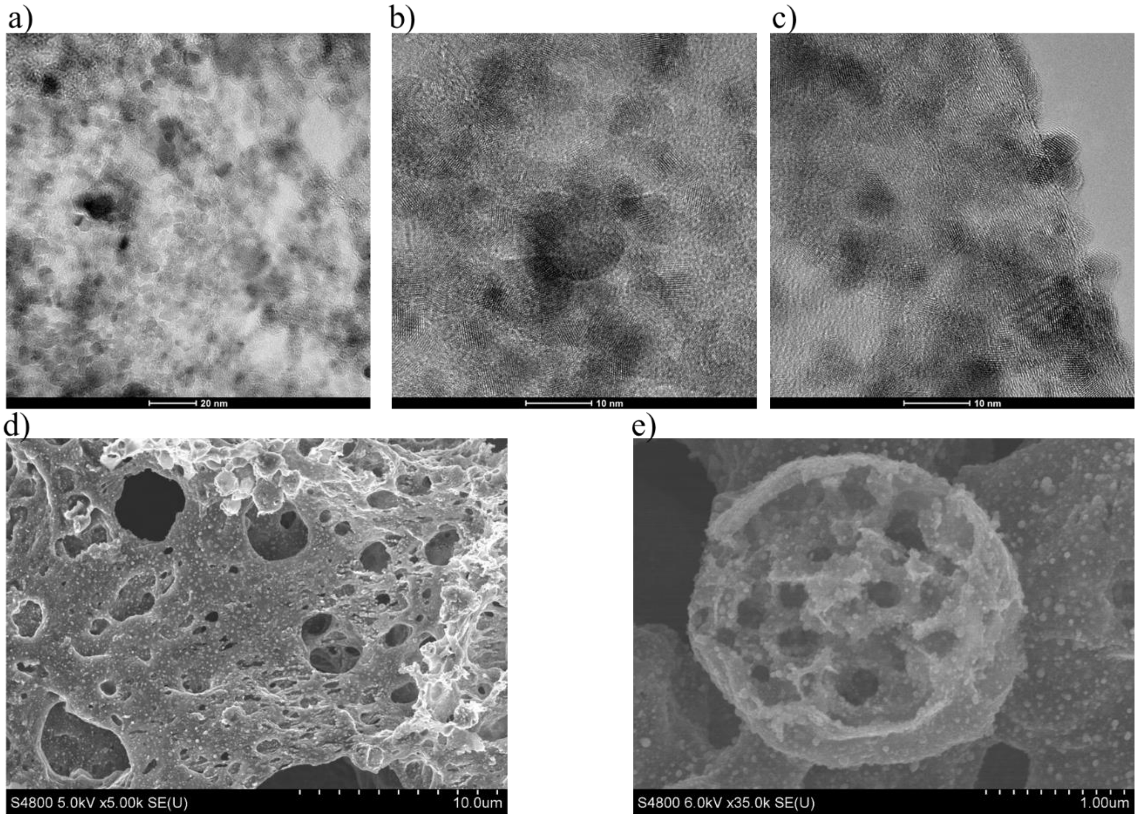

Figure S4. a, b, c) TEM and d) SEM images of ZIF67-13M.

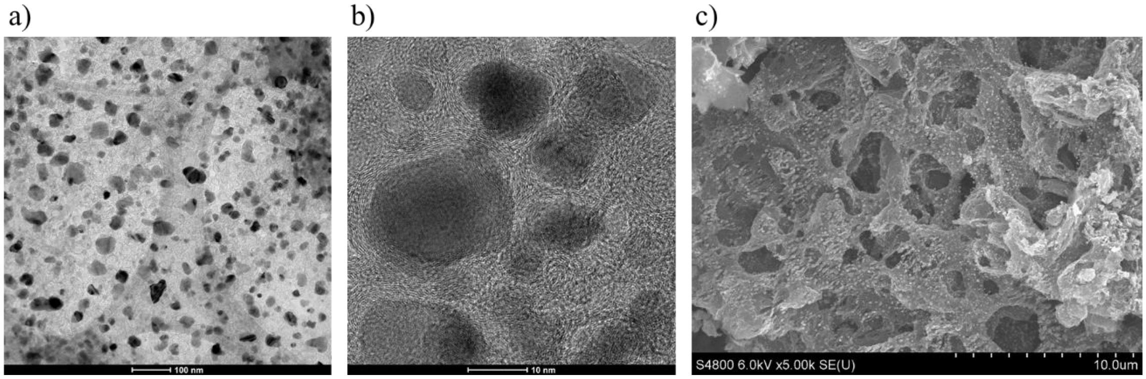

Figure S5. a, b) TEM and c) SEM images of ZIF67-11.

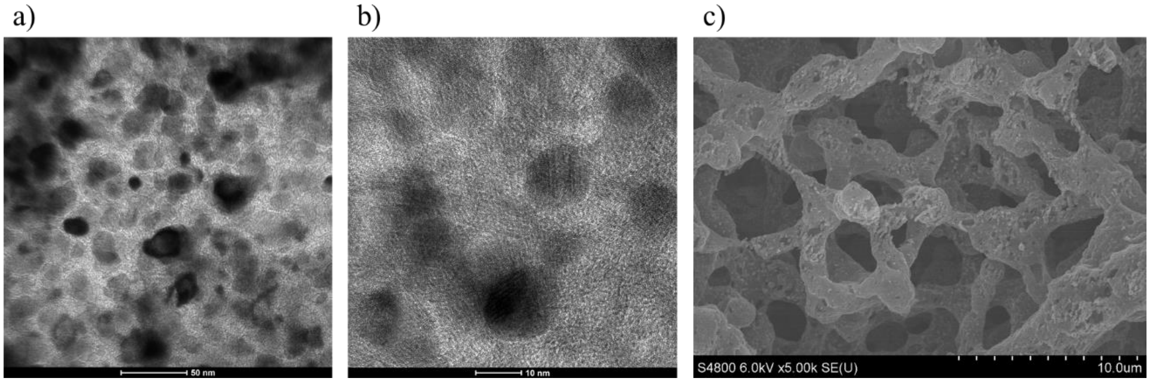

Figure S6. a, b) TEM and c) SEM images of ZIF67-12.

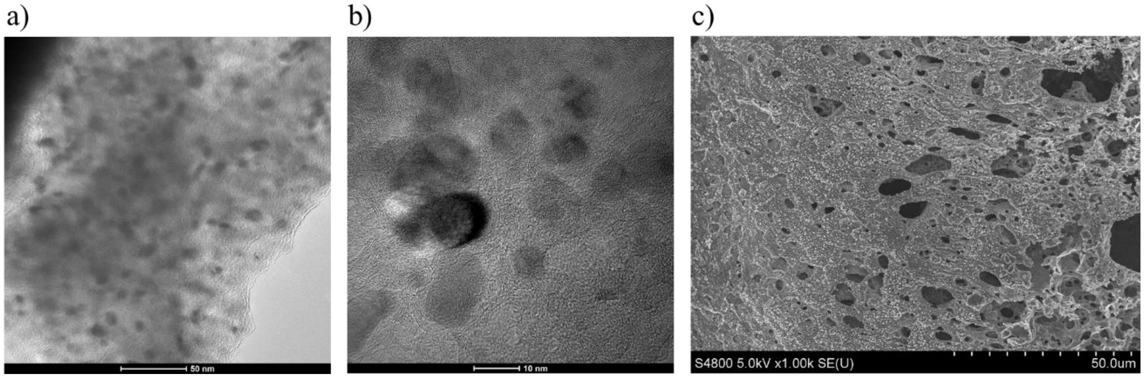

Figure S7. a, b) TEM and c) SEM images of ZIF67-13.

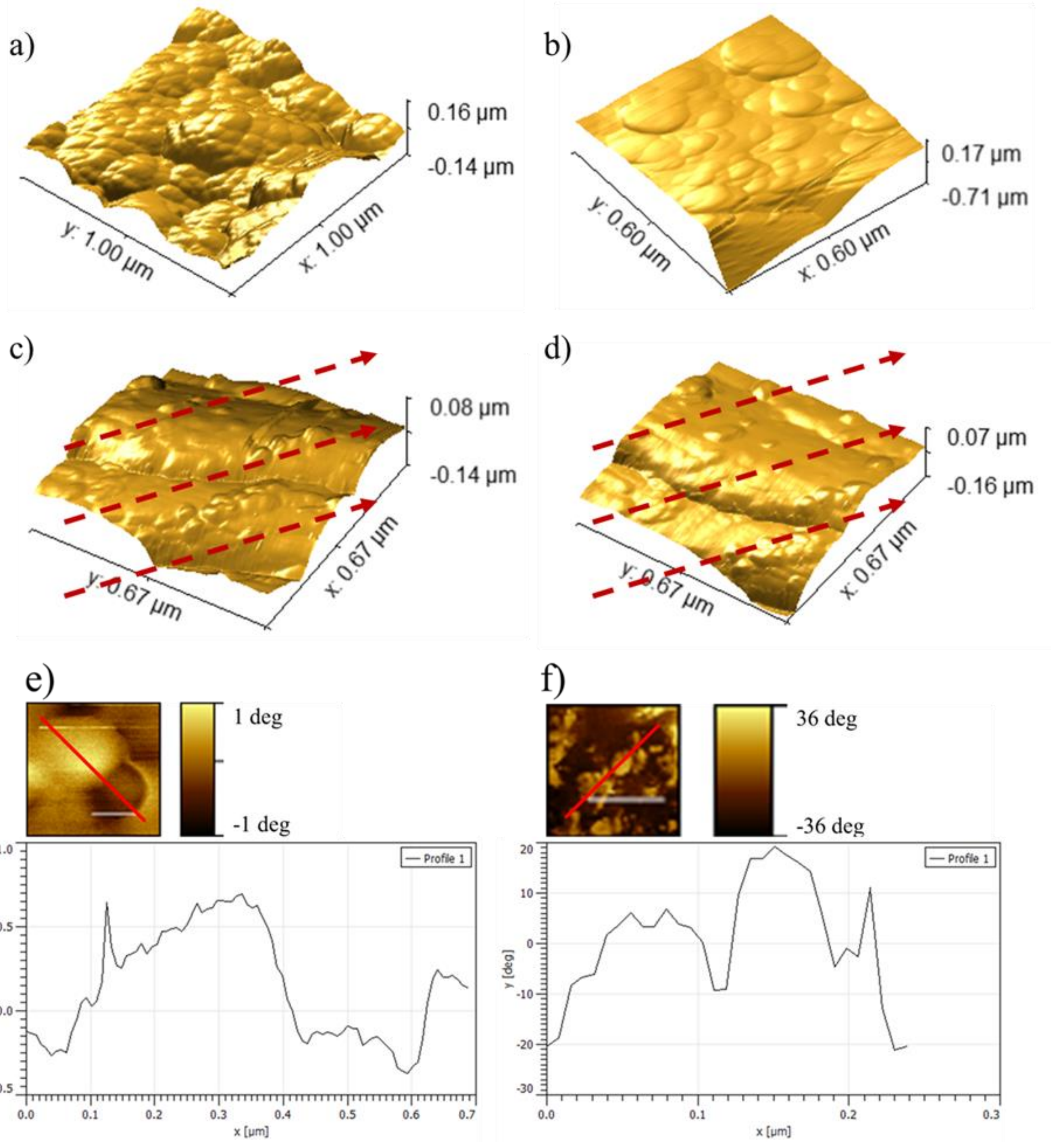

Figure S8. AFM images for a, b) ZIF67-13 and c, d) ZIF67-13M. The red arrows on c) and d) illustrate the aligned Co NPs along the MF. MFM line profiles for e) ZIF67-13 and f) ZIF67-13M.

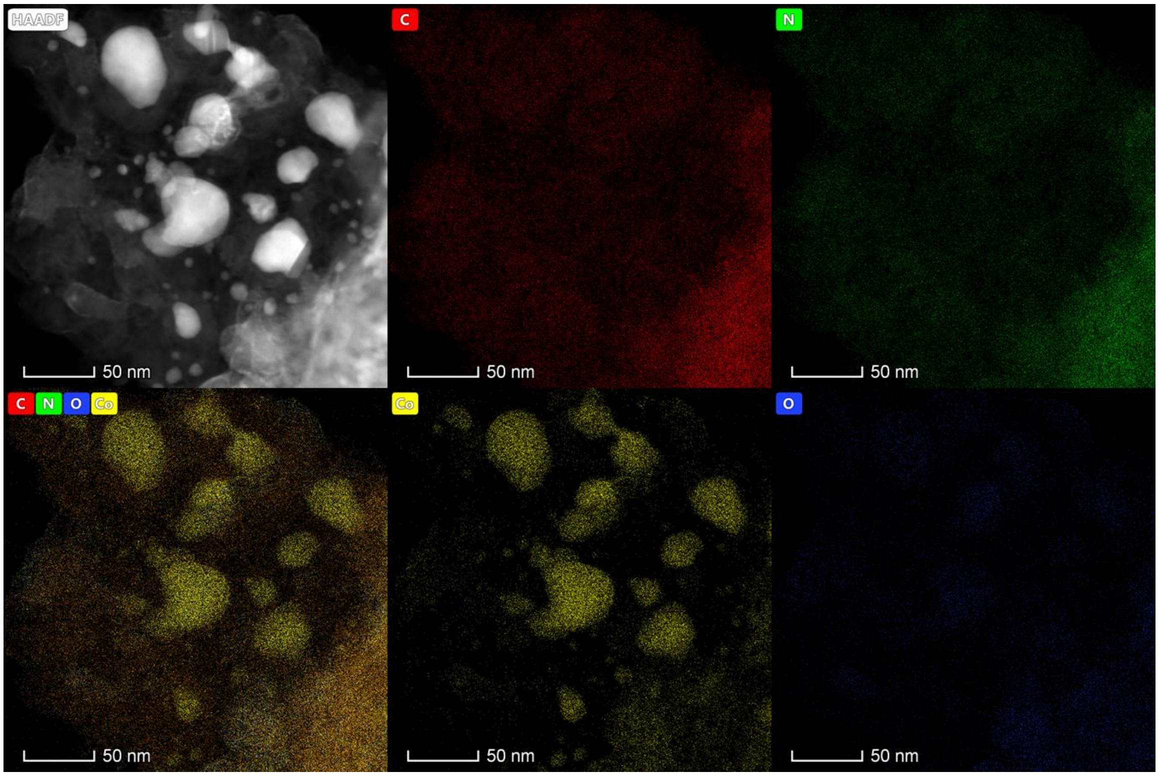

Figure S9. TEM EDS images for ZIF67-13M.

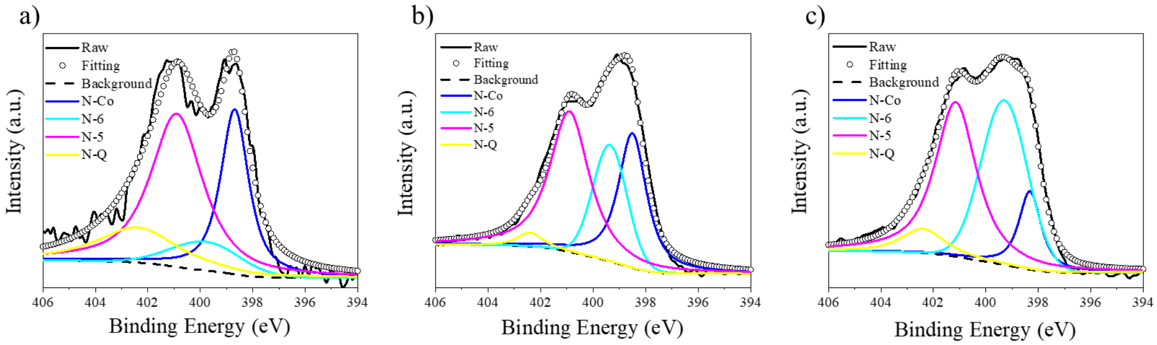

Figure S10. XPS N1s peaks for ZIF67 samples: a) ZIF67-A, b) ZIF67-13, c) ZIF67-13M.

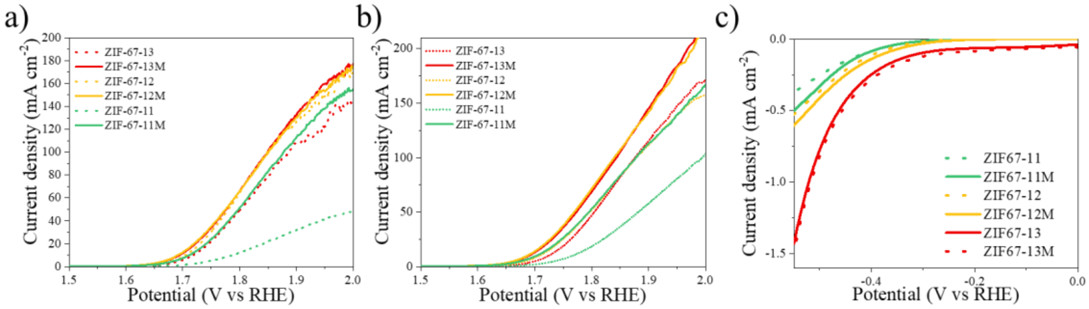

Figure S11. Electrocatalytic performance of samples. a) OER polarization curves for all samples under 10 mV s^{-1}, and b) those under 50 mV s^{-1}. c) HER polarization curves for all samples under 50 mV s^{-1}.

Laser	Without MF	With MF	ΔV (mV)
11	0.56	0.48	80
12	0.48	0.46	20
13	0.48	0.46	20

Table S1: OER Overpotentials under 10 mV s^{-1}.

Laser	Without MF	With MF	ΔV (mV)
11	0.50	0.47	30
12	0.47	0.45	20
13	0.54	0.45	90

Table S2: OER Overpotentials under 50 mV s^{-1}.

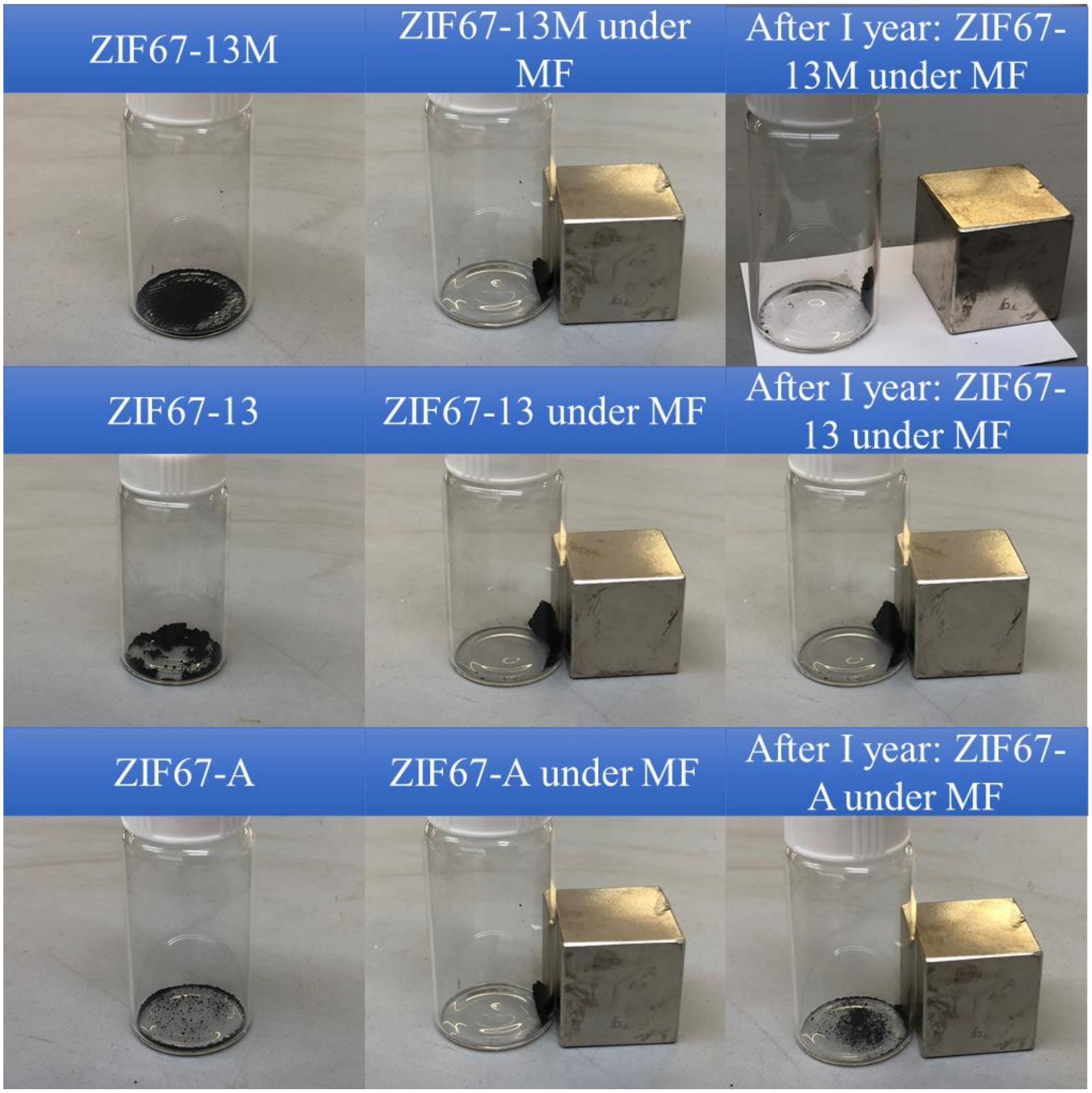

Figure S12. ZIF67-13, ZIF67-13M and ZIF67-A under external MF.

References

1] C. Ye, G. J. Cheng, S. Tao, B. Wu, Journal of Manufacturing Science and Engineering 2013, 135; K. S. Singh, A. K. Sharma, J Appl Phys 2016, 119, 183301; S. Tao, B. Wu, Y. Zhou, G. J. Cheng, Journal of Manufacturing Science and Engineering 2013, 135; B. Wu, S. Tao, Y.

Gao, Y. Zhou, G. Cheng, "The Effect of External Magnetic Field on the Plasma Induced by Laser Ablation in Vacuum", presented at *ASME 2011 International Mechanical Engineering Congress and Exposition*, 2011.
